# Expression changes of microRNA-1 and its targets Connexin 43 and brain-derived neurotrophic factor in the peripheral nervous system of chronic neuropathic rats

**DOI:** 10.1186/s12990-015-0045-y

**Published:** 2015-06-26

**Authors:** Elena Neumann, Henning Hermanns, Franziska Barthel, Robert Werdehausen, Timo Brandenburger

**Affiliations:** Department of Anesthesiology, Medical Faculty, Heinrich-Heine-University Düsseldorf, Moorenstr. 5, 40225 Düsseldorf, Germany; Department of Anesthesiology, Academic Medical Center, Meibergdreef 9, 1100 DD Amsterdam, The Netherlands

**Keywords:** microRNA, miR-1, Connexin 43 (Cx43), BDNF, Neuropathic pain, Chronic constriction injury (CCI)

## Abstract

**Background:**

MicroRNAs (miRNAs) are involved in the neuroplastic changes which induce and maintain neuropathic pain. However, it is unknown whether nerve injury leads to altered miRNA expression and modulation of pain relevant target gene expression within peripheral nerves. In the present study, expression profiles of miR-1 and the pain-relevant targets, brain derived neurotrophic factor (BDNF) and Connexin 43 (Cx43), were studied in peripheral neuropathic pain, which was induced by chronic constriction injury (CCI) of the sciatic nerve in rats. The expression of miR-1 was investigated in the sciatic nerve, dorsal root ganglion (DRG) and the ipsilateral spinal cord by qPCR. Changes of BDNF and Cx43 expression patterns were studied using qPCR, Western blot analysis, ELISA and immunohistochemistry.

**Results:**

In sciatic nerves of naïve rats, expression levels of miR-1 were more than twice as high as in DRG and spinal cord. In neuropathic rats, CCI lead to a time-dependent downregulation of miR-1 in the sciatic nerve but not in DRG and spinal cord. Likewise, protein expression of the miR-1 targets BDNF and Cx43 was upregulated in the sciatic nerve and DRG after CCI. Immunohistochemical staining revealed an endoneural abundancy of Cx43 in injured sciatic nerves which was absent after Sham operation.

**Conclusions:**

This study demonstrates that CCI leads to a regulation of miRNAs (miR-1) in the peripheral nervous system. This regulation is associated with alterations in the expression and localization of the miR-1 dependent pain-relevant proteins BDNF and Cx43. Further studies will have to explore the function of miRNAs in the context of neuropathic pain in the peripheral nervous system.

## Background

Neuropathic pain is caused by a lesion or disease of the somatosensory system involving alterations in the peripheral and the central nervous system [1]. The exact molecular mechanisms of neuropathic pain are incompletely understood and elucidation of these mechanisms is crucial for the development of new mechanism-oriented treatment strategies [2]. Neuroplastic changes in the peripheral and central nervous system, particularly alterations in protein expression in the pain processing neuronal network play a key role in the development of pathological pain [3].

MicroRNAs (miRNAs) are small non-coding RNAs which negatively regulate gene expression at the post-transcriptional level and have significant impact on numerous physiological and pathophysiological cellular processes [4]. MiRNA-targeting molecules are considered as possible future therapeutics for a variety of human diseases [5]. The fact that more than 60% of all human protein-coding genes are putative targets of miRNAs [6] suggests that miRNAs are correspondingly involved in the expression changes in chronic pain states. Increasing evidence suggests a significant role of non-coding RNAs, especially miRNAs, in the pathophysiology and potential treatment options of chronic pain [7]. A possible role of miRNAs in the development of chronic pain has to date been investigated in dorsal root ganglia, the spinal cord or in supraspinal organs [8]. One of the miRNAs being involved in neuropathic pain is miR-1. This miRNA has been shown to be involved in the induction of neuropathic pain [9]. Additionally, miR-1 interacts with the two highly pain-relevant proteins Cx43 and BDNF [10, 11]. It is well known that a multitude of neuroplastic alterations also occur in the peripheral nerve [12]. Furthermore, miRNAs are well abundantly expressed in the peripheral nerve, e.g. in Schwann cells but also in dendrites and axons, where they have been shown to be significantly regulated in response to peripheral nerve injury [13].

However, to date there is not data exploring the expression of miRNAs and pain relevant miRNA-target proteins in the context of neuropathic pain in peripheral nerves.

In this study we show that miR-1 is well expressed in sciatic nerves of rats. Furthermore we show that constriction injury of the sciatic nerve leads to a time dependent downregulation of miR-1 in injured nerves. This is accompanied by an upregulated protein expression of Connexin 43 (Cx43) and brain derived neurotrophic factor (BDNF) which are well established miR-1 targets.

## Results

### Development of neuropathic pain in rats

**Figure 1:**
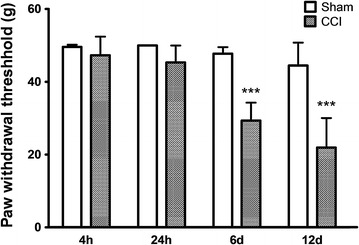
In vivo data on mechanical allodynia induced by chronic constriction injury (CCI) of the sciatic nerve. Paw withdrawal threshold of Sham (*left*) and CCI (*right*) animals at four different time points (*left injured paw*). Significant allodynia is observed on day 6 and 12 post CCI surgery. Mean ± SD, ***p < 0.001 vs. Sham.

Mechanical allodynia developed within 6 days after nerve ligation. In the left, injured hind paw, the withdrawal threshold in response to stimulation with von Frey hairs was not altered 4 h (Sham 49.6 ± 0.6 g, CCI 47.3 ± 5.1 g, p = 0.26) and 24 h (Sham 50.0 ± 0.1 g, CCI 45.3 ± 4.6 g, p = 0.06) after CCI. On day 6 (Sham 47.7 ± 1.8 g, CCI 29.3 ± 5.1 g, p = 0.0008) and day 12 (Sham 44.5 ± 6.3 g, CCI 21.9 ± 8.1 g, p = 0.0007) post CCI, the withdrawal thresholds were significantly reduced when compared to Sham-operated animals (Figure 1).

### Expression of miR-1 and Cx43 and BDNF messenger RNA

**Figure 2:**
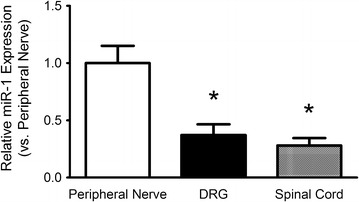
qPCR data demonstrating the expression of miR-1 in sciatic nerve (*left*), dorsal root ganglion (DRG, *middle*) and ipsilateral spinal cord (*right*) of naive rats. The expression of miR-1 is higher in peripheral nerve compared to DRG and spinal cord. Mean ± SD, *p < 0.05 vs. peripheral nerve.

The relative expression of miR-1 in sciatic nerve was compared to miR-1 expression in DRG and ipsilateral spinal cord of naïve rats. Expression level of miR-1 was higher in sciatic nerves than in DRG (relative expression DRG vs. nerve 0.37, p < 0.05) and the spinal cord (relative expression spinal cord vs. nerve 0.28, p < 0.05, Figure 2).

**Figure 3:**
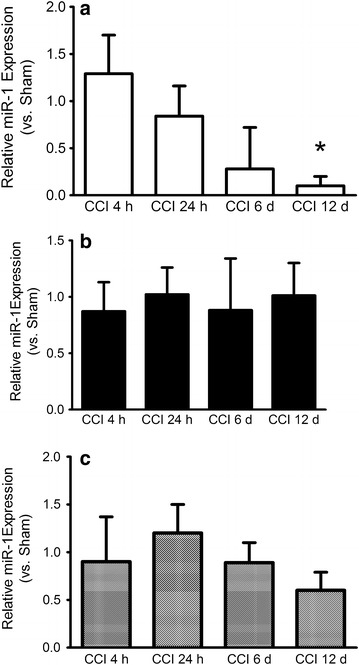
qPCR showing the time course of miR-1 expression following CCI in **a** sciatic nerve, **b** DRG, **c** ipsilateral spinal cord. Expression of miR-1 is time dependently downregulated in sciatic nerve and unchanged in DRG and spinal cord. Mean ± SD, *p < 0.05 vs. Sham.

In the sciatic nerve CCI lead to a marked downregulation of miR-1 12 days after nerve ligation (0.10 vs. Sham, p < 0.05, Figure 3a). After 6 days, the expression level of miR-1 in sciatic nerve was 0.28 vs. Sham but did not reach a level of significance (p = 0.053). Likewise, at the early time points after CCI surgery, miR-1 was not significantly altered in sciatic nerves (4 h: 1.29 vs. Sham, p = 0.6, 24 h: 0.84 vs. Sham, p = 0.72). In DRG and ipsilateral spinal cord, the expression level remained unchanged at all time points analyzed (Figure 3b, c).

**Figure 4:**
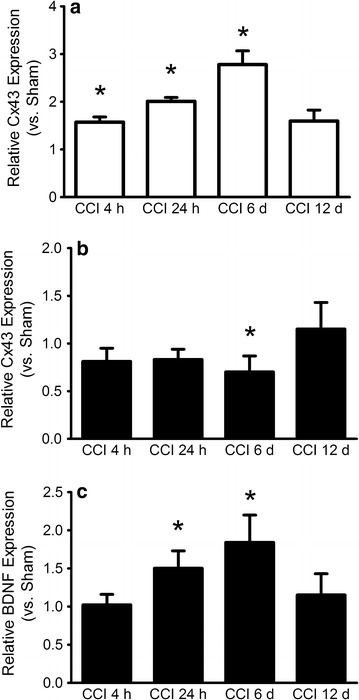
qPCR data of Cx43 mRNA expression in **a** peripheral nerve, where Cx43 is differentially expressed at three different time points and **b** DRG, where Cx43 is downregulated 6 days after CCI. **c** DRG: qPCR data of BDNF mRNA expression shows an upregulation 24 h and 6 days post CCI. Mean ± SD, *p < 0.05 vs. Sham.

The expression of Cx43 mRNA in the sciatic nerve was increased 4 h (relative expression 1.57 vs. Sham, p < 0.05), 24 h (relative expression 2.00 vs. Sham, p < 0.05) and 6 days (relative expression 2.78 vs. Sham, p < 0.05) post CCI surgery. After 12 days (relative expression 1.59 vs. Sham, p = 0.139) the relative expression of Cx43 mRNA declined to a not significant level (Figure 4a) compared to early expression. mRNA levels of BDNF in sciatic nerves of Sham-treated animals could not be detected using qPCR. In injured nerves of CCI rats however, mRNA of BDNF was detectable (data not shown).

In DRG, Cx43 mRNA expression remained unchanged 4, 24 h and 12 days post CCI. Six days after nerve ligation, the expression of Cx43 mRNA was reduced to 0.7-fold vs. Sham (p < 0.05). BDNF mRNA was upregulated in CCI DRG after 24 h (1.5-fold vs. Sham, p = 0.002), 6 days (1.84-fold vs. Sham, p = 0.018) and 12 days (1.58-fold vs. Sham, p = 0.007) post surgery.

### Protein expression of the miR-1 targets Cx43 and BDNF

**Figure 5:**
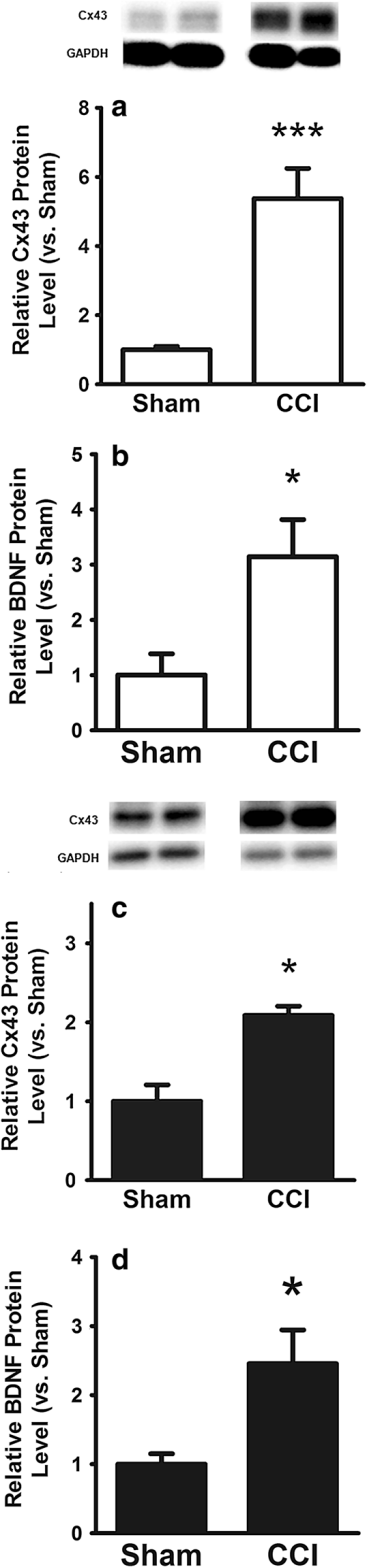
**a** Protein expression of Cx43 in sciatic nerve; **b** expression of BDNF protein in sciatic nerve; **c** protein expression of Cx43 in DRG; **d** expression of BDNF protein in DRG. All experiments compare rats 12 days after CCI to Sham. *Data* show a marked upregulation of Cx43 and BDNF protein levels in nerve and DRG following CCI. Mean ± SD, *p < 0.05 vs. Sham, ***p < 0.001 vs. Sham.

Cx43 and BDNF are well known targets of miR-1. We therefore analyzed the protein expression of Cx43 and BDNF in sciatic nerves and DRG. Cx43 was significantly upregulated in the ipsilateral sciatic nerve (5.4 fold vs. Sham, p < 0.01) and in L4–L6 DRG of the injured side (2.1 fold vs. Sham, p < 0.05) (Figure 5a, c). Likewise, ELISA showed a significant increase of BDNF protein levels in both the sciatic nerve (3.1 fold vs. Sham, p < 0.01) and DRG (2.5 fold vs. Sham, p < 0.01) of the injured side (Figure 5b,d).

### Immunofluorescence staining of Cx43

**Figure 6:**
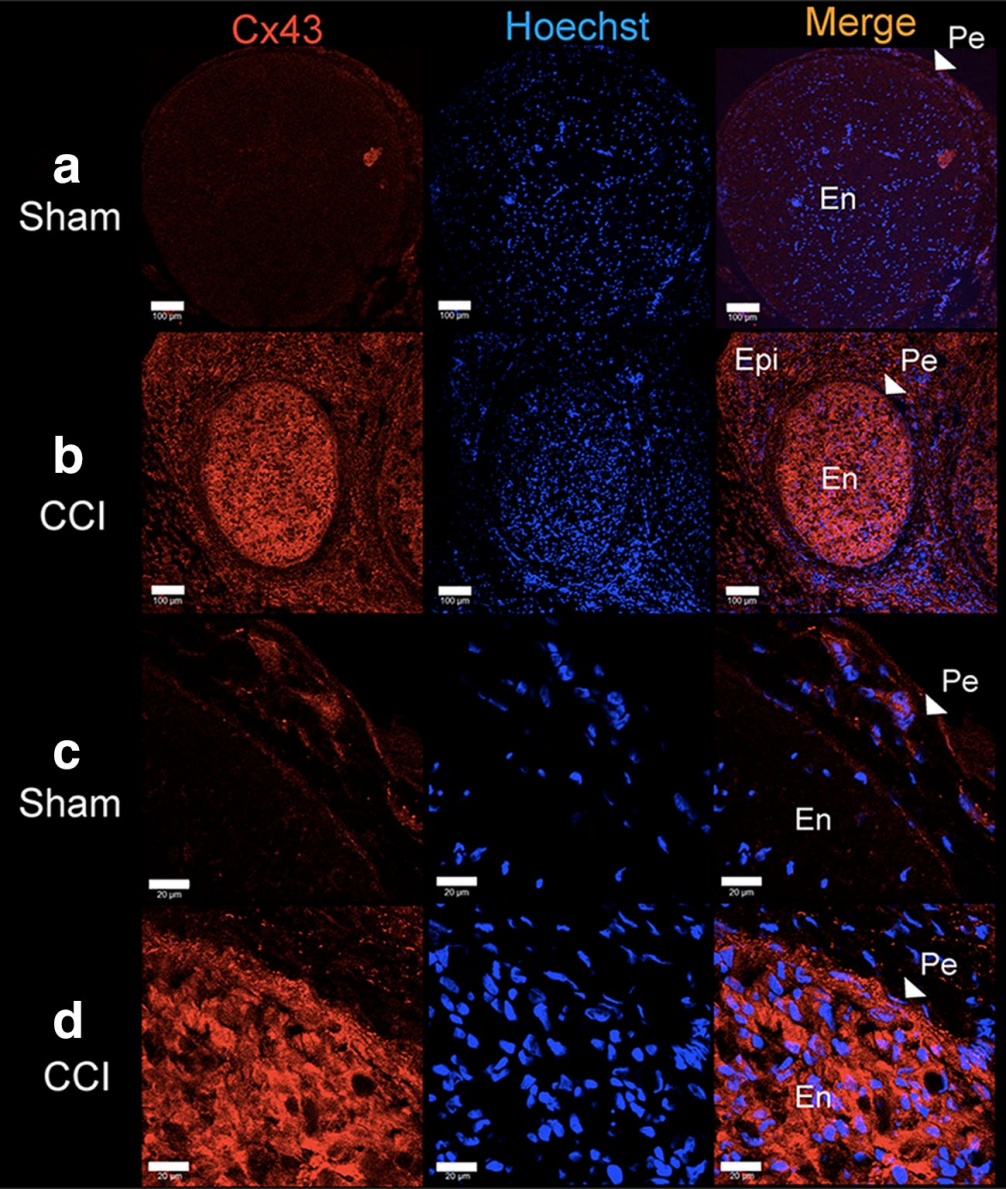
Immunohistochemical staining of Cx43 in sciatic nerve at lower (**a**, **b**, *scale bar* = 100 µm) and higher (**c**, **d**, *scale bar* = 20 µm) magnification. Expression of Cx43 in Sham nerves (**a**, **c**) is very faint and exclusively located at the perineural region. 12 days after CCI Cx43 is abundantly expressed within the endoneurium of the sciatic nerve (**b**, **d**). *En* endoneurium, *PE* perineurium, *Epi* epineurium, *Hoechst* nuclear counterstain.

**Figure 7:**
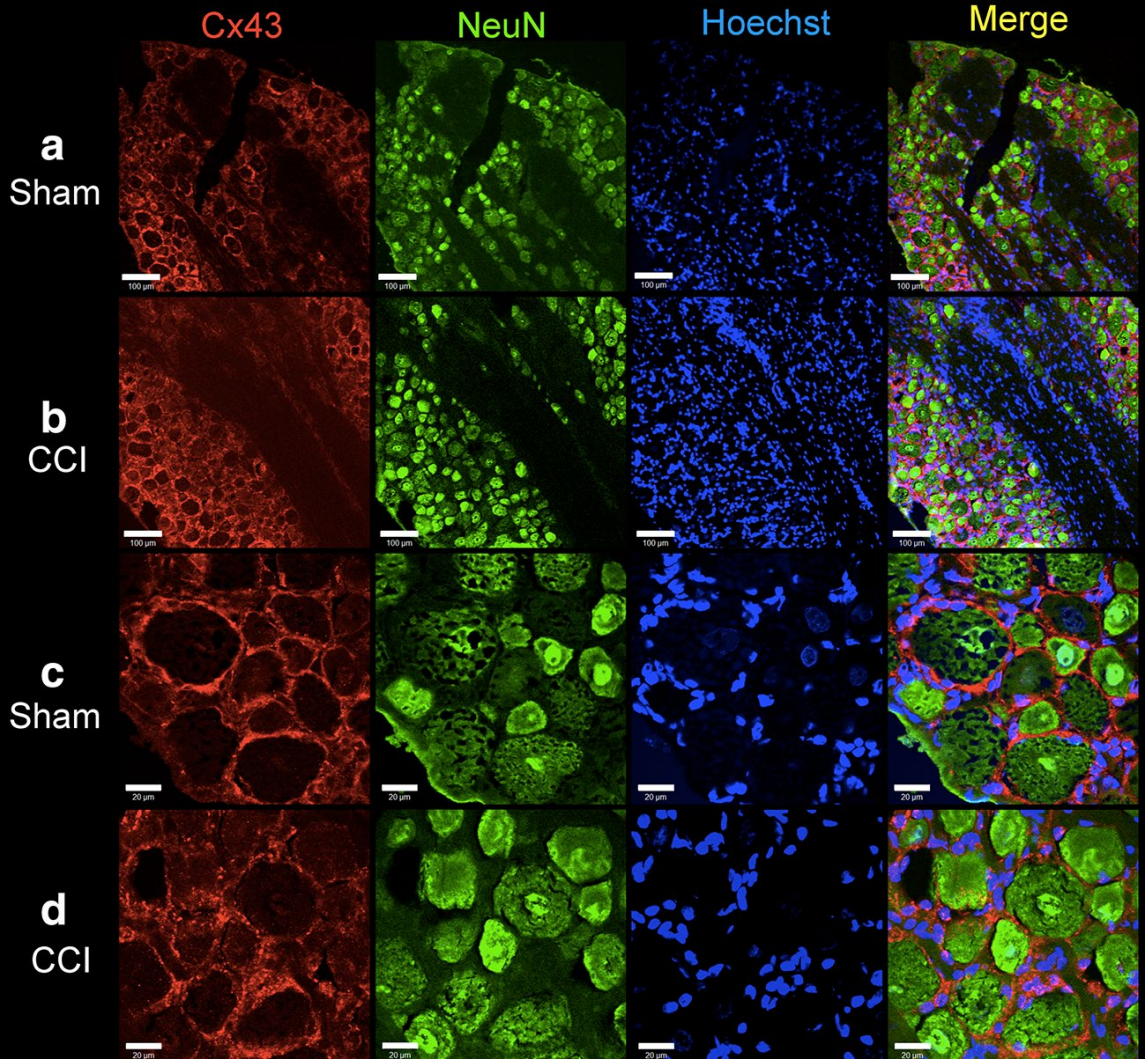
Immunohistochemical staining of Cx43 in DRG at lower (**a**, **b**, *scale bar* = 100 µm) and higher (**c**, **d**, *scale bar* = 20 µm) magnification. Cx43 is located at the plasma membrane of large and small diameter neurons in Sham (**a**, **c**) and CCI (12 days) rats (**b**, **d**). *NeuN* neuronal cell marker, *Hoechst* nuclear counterstain.

Immunofluorescence staining and confocal laser scanning microscopy were performed to show the subcellular localization of Cx43 in sciatic nerves and DRG of CCI and Sham treated animals. While in nerve tissue obtained from Sham animals Cx43 was located exclusively in the perineural region, a strong Cx43 signal predominately in the endoneurium of nerves was detectable (Figure 6) which is not apparent in Sham nerves. DRG sections show that Cx43 is located in the membranes of small and large diameter neurons with no apparent difference between Sham and CCI DRG’s (Figure 7).

## Discussion

To date, treatment of neuropathic pain remains challenging and often insufficient [14]. Elucidating the exact molecular mechanisms underlying neuropathic pain is an important prerequisite for developing more specific and more efficient treatment strategies [2]. In the last few years, the involvement of miRNAs in neuropathic pain has been investigated. MiRNAs are involved in the development of the nervous system, neural plasticity and the genesis of neuronal diseases [15]. In 2009, Aldrich et al. showed for the first time a downregulation of miR-96, -182 and -183 in DRG of rats in the context of neuropathic pain [16]. Since then, numerous studies concerning the role of miRNAs in experimental neuropathic pain have been published [17–19]. Several studies have furthermore shown that the application of miRNAs or its antagonists has an antinociceptive effect in various animal models. These include miR-103 [18], miR-124 [20], miR-23b [21] and miR-7a [22]. Beyond that, a role of extracellular miRNAs in pain signalling via direct activation and excitation of nociceptor neurons has been proposed recently [23]. Hence, the potential for miRNAs as biomarkers or therapeutics in chronic pain is now being discussed extensively [7, 8, 24–28]. The role of miRNAs in the peripheral nerve in the context of neuropathic pain has however not been explored, although it is well known that massive protein expression changes accompany nerve damage [12]. In general, the contribution of miRNAs to the regulation of gene expression in peripheral nerves is well recognized, especially in the context of regenerative processes [13]. Viader et al. [29] identified 87 miRNAs in the sciatic nerve of mice, including mir-1, most of which were downregulated upon peripheral nerve injury. Since Schwann cells represent the vast majority of cells in the sciatic nerve, the authors concluded that the detected miRNAs primarily represent the microRNAome of these glia cells. Yet, a few studies also demonstrated the abundance of numerous mature miRNAs in axons [30], whereby the functional significance in axonal mRNA translation remains to be elucidated.

In the present study we could demonstrate that miR-1 is expressed in the sciatic nerve of rats. Its expression level was found to be higher in sciatic nerve than in DRG or spinal cord. Furthermore, as a consequence of CCI leading to mechanical allodynia, we found miR-1 to be time-dependently downregulated in the sciatic nerve at the site of the constriction, whereas expression in the ipsilateral DRG and spinal cord remained unchanged. BDNF, a previously established target of mir-1 [11] was significantly upregulated in the sciatic nerve and DRG. Similarly, connexin-43, another established miR-1 target [10] was upregulated in peripheral nerves and DRG.

Kusuda and co-workers [9] investigated the expression of mir-1 in the DRG and spinal cord in different experimental models of acute and chronic pain. After partial sciatic nerve ligation, a model of neuropathic pain, miR-1 was time-dependently down-regulated in the DRG. Expression of miR-1 in acute, inflammatory pain, as well as axotomy revealed time- and stimulus-dependent changes in expression patterns which underline the complexity of expression changes in different pain states.

Our results obtained in the spinal cord are in line with those described by Kusuda et al. In contrast, we found no changes of miR-1 expression in the DRG, which might result from the differences in species or surrogate model of neuropathic pain.

The fact that BDNF is upregulated in Schwann cells of peripheral nerves as response to neuronal damage is long known [31], as is the contribution of Schwann cell-mediated responses to nerve injury in neuropathic pain [32]. Also the effect of BDNF as an ubiquitous pain mediator in the nervous system is sufficiently documented, although its action in the peripheral system is less extensively investigated [33]. These results are in line with our observation that mRNA of BDNF is absent in Sham sciatic nerves, while it is detectable in injured nerves, most likely as a consequence of expression by Schwann cells.

The expression of BDNF in our study could at least in part be explained by an upregulation in DRG neurons independently of miRNAs. However, the dysregulation of miR-1 expression in injured nerves could at least in part be involved in the expression of Cx43 in nerves. Interestingly, Cx43 is highly upregulated in the endoneural region of nerves where it might contribute to an increased electrochemical coupling of cells contributing to the induction and/or maintenance of pain. Cx43 is the major connexin of astrocytes [34]. A recent study by Chen et al. showed that the upregulation of spinal Cx43 contributes to the maintenance of neuropathic pain by chemokine release. Moreover, inhibition of Cx43 by intrathecal injection of GAP27, a selective Cx43-blocker, effectively reduced mechanical allodynia in CCI mice [35]. To the best of our knowledge, the present study is the first to show a marked upregulation of Cx43 within the endoneurium of injured nerves which might—besides a transcriptional activation of Cx43 expression—in part be explained by a downregulation of miR-1. This differential regulation of miR-1 in the peripheral nerve, most likely in Schwann cells, might contribute to hypersensitivity following nerve damage. However, also the transcriptional activation of Cx43 mRNA might contribute to the protein changes observed in nerves after CCI and further studies are needed to clarify the impact of the miR-1 dysregulation in nerves.

## Conclusion

In summary, our findings suggest an involvement of regulated miR-1 in the peripheral nerve in neuropathic pain. If these findings are confirmed in future studies, a local therapy with compounds regulating miR-1 or other relevant miRNAs could be a theoretical approach for the treatment of neuropathic pain. Ultimately, future research on the involvement of miRNAs in the development of neuropathic pain should also consider the contribution of miRNAs in the peripheral nervous system.

## Methods

### Animal experiments

The study was conducted with male Wistar rats (weight 300–350 g, 10–12 weeks old) after approval of the local animal care and use committee (LANUV). Neuropathic pain was induced by the chronic constriction injury (CCI) model [36] as described previously [37]. Sham-operated animals served as controls. The development of mechanical allodynia was assessed with modified von Frey hairs (Plantar Aesthesiometer, Ugo Basile Inc., Comerio, Italy) before initial surgery and at every time point before tissue extraction. The withdrawal threshold as response to mechanical stimulation was registered. At the end of the experiment, the left sciatic nerve (1 cm including the site of the ligations), lumbar DRG (L4–L6) and the ipsilateral spinal cord (L4–L6) were extracted 4, 24 h, 6 and 12 days after CCI or Sham operation and immediately frozen in liquid nitrogen (n = 6 each).

### RNA isolation

Total RNA of sciatic nerve, DRG and spinal cord tissue was isolated using Trizol reagent (Invitrogen, Carlsbad, USA) according to the manufacturer’s protocol. RNA quantity was determined by UV spectrophotometry (Nanodrop 1000, Thermo Scientific, Waltham, MA, USA) and RNA integrity was verified by Agilent microfluid chips using an Agilent 2100 Bioanalyzer (Agilent, Santa Clara, CA, USA). Only RNA with a RNA integrity number (RIN) higher than 8 was included in the study.

### Realtime quantitative PCR

1 µg of total RNA was reverse transcribed using the High Capacity RNA-to-cDNA Master Mix (Applied Biosystems, Life Technologies, Carlsbad, CA, USA). qPCR assays for rno-miR-1 (Assay ID: 002064, Applied Biosystems), U6 (for normalization, Assay ID: 001973, Applied Biosystems), BDNF (Assay ID: Rn02531967_s1) and actin beta (Rn00667869_m1) were applied according to the manufacturer’s instructions. The qPCR assay for Cx43 was designed by and purchased from TIB Molbiol (Berlin, Germany) and has the following sequences: Primer_for: AGGAGTTCCACCAACTTTGGC, Primer_rev: TGGAGTAGGCTTGGACCTTGTC and Taqman probe: FAM-AGCTTCCCCAAGGCACTCCAGTC-BBQ. qPCR conditions: 50°C for 2 min, 95°C for 10 min, 40 cycles of 95°C for 15 s, 60°C for 60 s on an Applied Biosystems 7300HT thermocycler (Applied Biosystems). All samples were run in duplicates. Relative expression was estimated using the ΔΔCq-method [38] and the relative expression software tool [39].

### Protein isolation and Western Blot experiments

To analyze Cx43 protein expression in the sciatic nerve and DRG, samples were pulverized in liquid nitrogen, homogenized in a buffer of pH 8.0 (50 mM Tris, 150 mM NaCl, 1% NP40, 0.5% Na-Deoxycholate, 0.1% SDS, 40 μl/ml Complete) and centrifuged at 4°C, 8,000*g* for 10 min. The supernatant was harvested and the protein content was measured according to Lowry et al. Sodium dodecyl sulfate polyacrylamide gel electrophoresis separated equal amounts of protein (40 μg per lane). The gel was run for 85 min at 100 V and the proteins were transferred to a polyvinylidene difluoride membrane at 220 mA for 1 h. The membrane was blocked with 5% dried skimmed milk in Tris-buffered saline with 0.1% Tween for 2 h at room temperature and incubated with the primary antibody (Cx43, ab11370, abcam, Cambridge, UK, 1:1,000) overnight at 4°C. After washing with cold TBS-T three times for 10 min the secondary antibody was applied for 2 h. The membrane was washed again in TBS-T and bound antibodies were visualized using the enhanced chemoluminescent detection method by a digital camera (cool snap HQ2; Photometrics^®^, Tuscon, AZ). Signals were quantified and standardized against GAPDH (Abcam ab8245, Cambridge, UK) by densitometry (GelScan; BioSciTec GmbH, Frankfurt/Main, Germany).

### ELISA

BDNF protein levels of sciatic nerve and DRG were analysed using the BDNF Emax^®^ ImmunoAssay System (Promega, Madison, WI, USA) according to the manufacturer’s protocol as described previously [40]. Briefly, 96-well plates were coated with anti-BDNF monoclonal antibody and incubated at 4°C overnight. The plates were washed with TBS-T and incubated with 200 μl of Block and Sample buffer (BDNF E_max_™ ImmunoAssay System, Promega, Madison, WI, USA) for 1 h at room temperature. 100 μl of each sample prepared as described above was transferred to the anti-BDNF-coated ELISA plate. BDNF levels were determined from the standard curve prepared for each plate. The standard curves were linear within the range used (0–500 pg/ml).

### Immunohistochemical staining and confocal microscopy

DRG and nerve tissue was removed and embedded in Tissue -Tek^®^ O.C.T. (Sakura Finetek Europe, Alphan aan den Rijin, The Netherlands) in cryomolds at −40°C without prior treatment and stored at −20°C. Cryosections of rat DRG and nerve tissue (8 µm) were fixed in 4% PFA containing 0.1 M phosphate buffer for 10 min and washed in 3× PBS for 10 min. Slices were blocked with 10% goat serum in PBS containing 0.2% saponin. Cross sections of sciatic nerves were incubated with Cx43 (Cx43, ab11370, abcam, Cambridge, UK, 1:1,000), DRG sections were incubated with Cx43 and NeuN antibodies (Anti-NeuN, clone A60, Cat. # MAB377, Millipore, Temecula, USA) at 4°C overnight. After washing with PBS/saponin, slices were incubated with Cy3-labeled anti-rabbit IgG (Lot number 81,350, Dianova, Hamburg, Germany, 1:500) and Alexa Fluor 488 conjugated goat anti-mouse IgG (A-11029, Life Technologies Carlsbad, USA, 1:500) containing Hoechst 34580 (1:10,000) as nuclear counterstain for 1 h at room temperature. After washing with PBS/saponin, slices were mounted with FuoroMount G medium. Immunostained samples were analysed using a Zeiss LSM510META confocal microscope (Jena, Germany).

### Statistical analysis

Behavioral data, Western Blot data and BDNF ELISA results were analyzed by student’s t test (GraphPad Prism version 6, GraphPad Software, San Diego, CA USA). qPCR data was analyzed using the relative expression software tool [39]. Data are presented as mean ± SD. p < 0.05 was considered statistically significant.
